# Supplementary material for: Temperature and Host Fruit During Immature Development Shape Adult Life History Traits of Different Ceratitis capitata Populations
Source: Insects. 2025 Jan 11;16(1):65. doi: 10.3390/insects16010065 (PMC11765621; doi:10.3390/insects16010065)
Supplement: Supplementary file 1 [file insects-16-00065-s001.zip › insects-3386092-supplementary.pdf]

Supplementary Information

Supplementary Table S1: Number of *Ceratitis capitata* individuals by Population, Host Plant, and Acclimation Temperature used in the experiment

Population	Host	Acclimation_temperature(°C)	Number of individuals
Vienna	Apples	15	12
Zaton	Apples	15	-
Thessaloniki	Apples	15	28
Volos	Apples	15	42
Chios	Apples	15	10
Crete	Apples	15	14
Vienna	Apples	20	66
Zaton	Apples	20	32
Thessaloniki	Apples	20	70
Volos	Apples	20	48
Chios	Apples	20	36
Crete	Apples	20	54
Vienna	Apples	25	90
Zaton	Apples	25	78
Thessaloniki	Apples	25	56
Volos	Apples	25	46
Chios	Apples	25	36
Crete	Apples	25	58
Vienna	Bitter oranges	15	-
Zaton	Bitter oranges	15	2
Thessaloniki	Bitter oranges	15	26
Volos	Bitter oranges	15	22
Chios	Bitter oranges	15	24
Crete	Bitter oranges	15	2
Vienna	Bitter oranges	20	46
Zaton	Bitter oranges	20	46

Thessaloniki	Bitter oranges	20	70
Volos	Bitter oranges	20	44
Chios	Bitter oranges	20	78
Crete	Bitter oranges	20	40
Vienna	Bitter oranges	25	52
Zaton	Bitter oranges	25	46
Thessaloniki	Bitter oranges	25	75
Volos	Bitter oranges	25	40
Chios	Bitter oranges	25	42
Crete	Bitter oranges	25	80

Supplementary Table S2: Pairwise comparisons (Population * Population) of estimated marginal means based on Lifespan of *C. capitata* populations. The mean difference is significant at the level 0.05.

**Comparison groups**	**Mean Difference ± SE**	**df**	***p***
Zaton x Vienna	6.15 ± 3.14	1	0.750
Zaton x Thessaloniki	4.06± 2.80	1	1.000
Zaton x Volos	23.30± 3.25	1	<0.001
Zaton x Chios	17.16± 3.26	1	<0.001
Zaton x Crete	1.56±3.16	1	1.000

Supplementary Table S3: Effects of explanatory variables of the linear models on lifespan of *C. capitata* populations. Males from Crete reared in bitter oranges at 15°C form the baseline.

**Variables in the model**	**B ± SE**	**Wald x^2**	**df**	***p***
Intercept	49.105 ± 15.97	9.446	1	0.002
Host	-2.510 ± 1.59	2.478	1	0.115
Population				<0.001
Vienna	44.634 ± 21.34	4.372	1	0.037
Zaton	41.669 ± 25.04	2.768	1	0.096
Thes/niki	-46.152 ± 17.72	6.780	1	0.009
Volos	-41.364 ± 18.58	4.952	1	0.026
Chios	-40.559 ± 20.20	4.031	1	0.045
Females	-1.729 ± 11.42	0.023	1	0.880
Temperature	1.386± 0.69	3.962	1	0.047
Population x Sex				<0.001
Vienna x female	-12.563 ± 5.61	5.002	1	0.025
Zaton x female	-25.016 ± 6.02	17.255	1	<0.001
Thes/niki x female	-2.173 ± 4.95	0.192	1	0.661
Volos x female	-0.568 ± 5.83	0.009	1	0.922
Chios x female	-10.611 ± 5.89	3.236	1	0.072
Population x Temperature				
Vienna x Temperature	-1.965 ± 0.93	4.420	1	0.036
Zaton x Temperature	-1.263± 1.08	1.362	1	0.243
Thes/niki x Temperature	2.046 ± 0.78	6.898	1	0.009
Volos x Temperature	0.911 ± 0.84	1.186	1	0.276
Chios x Temperature	1.384± 0.91	2.325	1	0.127
Sex x Temperature				0.294

Supplementary Table S4: Pairwise comparisons (Population * Sex) of estimated marginal means based on Lifespan of *C. capitata* populations. The mean difference is significant at the level 0.05.

Comparison groups	Mean Difference ± SE	df	*P*
Vienna females x Vienna males	-25.22 ± 3.91	1	<0.001
Zaton females x Zaton males	-37.67± 4.49	1	<0.001
Thes/nik females x Thes/niki males	-14.83± 2.86	1	<0.001
Volos females x Volos males	-13.22± 4.15	1	0.097
Chios females x Chios males	-23.27±4.26	1	<0.001
Crete females x Crete males	-12.65 ± 4.04	1	0.116

Supplementary Table S5: Pairwise comparisons (Population * Population) of estimated marginal means based on Lifespan of *C. capitata* populations. The mean difference is significant at the level 0.05.

Comparison groups	Mean Difference ± SE	df	*p*
Thes/niki x Vienna	9.39 ± 1.65	1	<0.001
Thes/niki x Zaton	19.03± 1.74	1	<0.001
Thes/niki x Volos	6.73± 1.84	1	0.004
Thes/niki x Chios	6.32± 1.92	1	0.015
Thes/niki x Crete	17.88± 1.68	1	<0.001

Supplementary Table S6: Pairwise comparisons (Population * Host) of estimated marginal means based on Pre oviposition period of *C. capitata* populations. The mean difference is significant at the level 0.05.

Comparison groups	Mean Difference ± SE	df	*p*
Vienna Apples x Vienna Bitter oranges	-0.43± 2.61	1	1.000
Zaton Apples x Zaton Bitter oranges	-3.87± 2.81	1	1.000
Thes/nik Apples x Thes/niki Bitter oranges	11.76± 2.04	1	<0.001
Volos Apples x Volos Bitter oranges	11.06± 3.06	1	0.020
Chios Apples x Chios Bitter oranges	9.76±3.25	1	0.180

Crete Apples*Crete Bitter oranges	-1.79 ± 2.69	1	1.000

Supplementary Table S7: Pairwise comparisons (Population * Population) of estimated marginal means based on Oviposition period of *C. capitata* populations. The mean difference is significant at the level 0.05.

Comparison groups	Mean Difference ± SE	df	*p*
Zaton **x** Vienna	6.00 ± 2.76	1	0.451
Zaton x Thessaloniki	10.84± 2.53	1	<0.001
Zaton x Volos	15.76± 3.06	1	<0.001
Zaton x Chios	16.63± 3.01	1	<0.001
Zaton x Crete	4.32±2.87	1	1.000

Supplementary Table S8: Effects of explanatory variables of the linear models on oviposition of *C. capitata* populations. Females from Crete reared in bitter oranges at 15°C form the baseline.

Variables in the model	B ± SE	Wald x^2	df	*p*
Intercept	63.238 ± 15.02	17.717	1	<0.001
Host	-3.170± 1.54	4.216	1	0.040
Population				<0.001
Vienna	-12.123± 20.80	0.340	1	0.560
Zaton	-25.11 ± 22.77	1.216	1	0.270
Thes/niki	-67.591 ± 17.80	14.404	1	<0.001
Volos	-56.047± 19.08	8.621	1	0.003
Chios	-61.400 ± 21.00	8.548	1	0.003
Temperature	-1.185± 0.65	3.227	1	0.072
Population x Temperature				
Vienna x Temperature	0.473 ± 0.91	0.265	1	0.606
Zaton x Temperature	1.332 ± 0.99	1.792	1	0.181
Thes/niki x Temperature	2.764 ± 0.78	12.269	1	<0.001

Volos x Temperature	2.019 ± 0.88	5.431	1	0.020
Chios x Temperature	2.222 ± 0.95	5.460	1	0.019

Supplementary Table S9: Pairwise comparisons (Population * Population) of estimated marginal means based on Post oviposition period of *C. capitata* populations. The mean difference is significant at the level 0.05.

Comparison groups	Mean Difference ± SE	df	*p*
Crete x Vienna	9.14 ± 2.09	1	<0.001
Crete x Zaton	9.21 ± 2.22	1	0.001
Crete x Thessaloniki	5.12 ± 1.87	1	0.095
Crete x Volos	8.87 ± 2.31	1	0.002
Crete x Chios	11.46± 2.36	1	<0.001

Supplementary Table S10: Pairwise comparisons (Population * Host) of estimated marginal means based on Post oviposition period of *C. capitata* populations. The mean difference is significant at the level 0.05.

Comparison groups	Mean Difference ± SE	df	*p*
Vienna Apples x Vienna Bitter oranges	4,33± 2.88	1	1.000
Zaton Apples x Zaton Bitter oranges	-1.66± 3.20	1	1.000
Thes/nik Apples x Thes/niki Bitter oranges	-5.13± 2.24	1	1.000
Volos Apples x Volos Bitter oranges	-2.64± 3.45	1	1.000
Chios Apples x Chios Bitter oranges	-1.45±3.66	1	1.000
Crete Apples x Crete Bitter oranges	28.78 ± 3.03	1	<0.001

Supplementary Table S11: Effects of explanatory variables of the linear models on Post oviposition of *C. capitata* populations. Females from Crete reared in bitter oranges at 15°C form the baseline.

Variables in the model	B ± SE	Wald x^2	df	*p*
Intercept	-74.569 ± 13.73	29.476	1	<0.001
Population				<0.001

Vienna	93.531± 16.86	30.743	1	<0.001
Zaton	91.770 ± 18.33	25.064	1	<0.001
Thes/niki	84.126 ± 14.63	33.049	1	<0.001
Volos	90.576 ± 15.91	32.379	1	<0.001
Chios	80.293 ± 17.30	8.548	1	0.003
Temperature	3.464 ± 0.58	34.868	1	<0.001
Host x Population				
Apples x Vienna	-24.451 ± 4.17	34.225	1	<0.001
Apples x Zaton	-30.443 ± 4.40	47.833	1	<0.001
Apples x Thes/niki	-33.913 ± 3.77	80.527	1	<0.001
Apples x Volos	-31.416 ± 4.62	46.134	1	<0.001
Apples x Chios	-30.239 ± 4.76	40.232	1	<0.001
Apples x Temperature	0.965 ± 0.39	5.987	1	0.014
Population x Temperature				
Vienna x Temperature	-4.093 ± 0.72	31.956	1	<0.001
Zaton x Temperature	-3.881 ± 0.79	23.673	1	<0.001
Thes/niki x Temperature	-3.271 ± 0.62	27.014	1	<0.001
Volos x Temperature	-3.789 ± 0.69	30.022	1	<0.001
Chios x Temperature	-3.468 ± 0.77	20.097	1	<0.001
